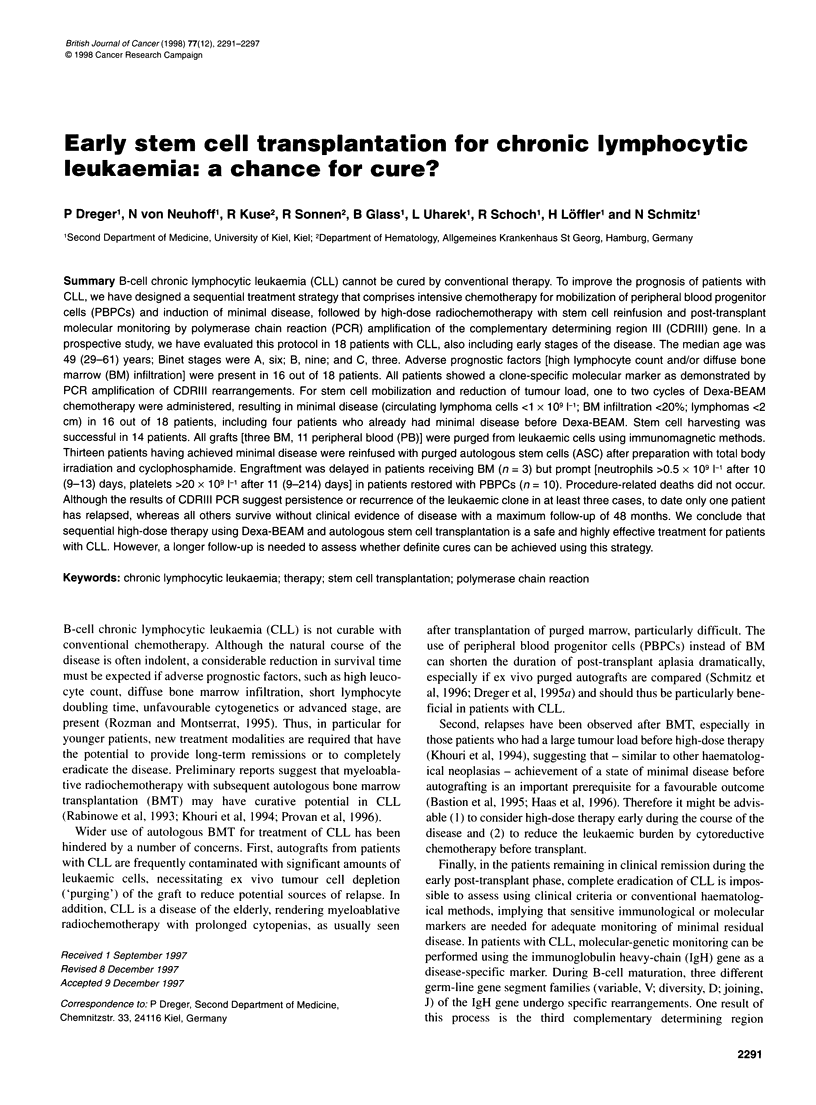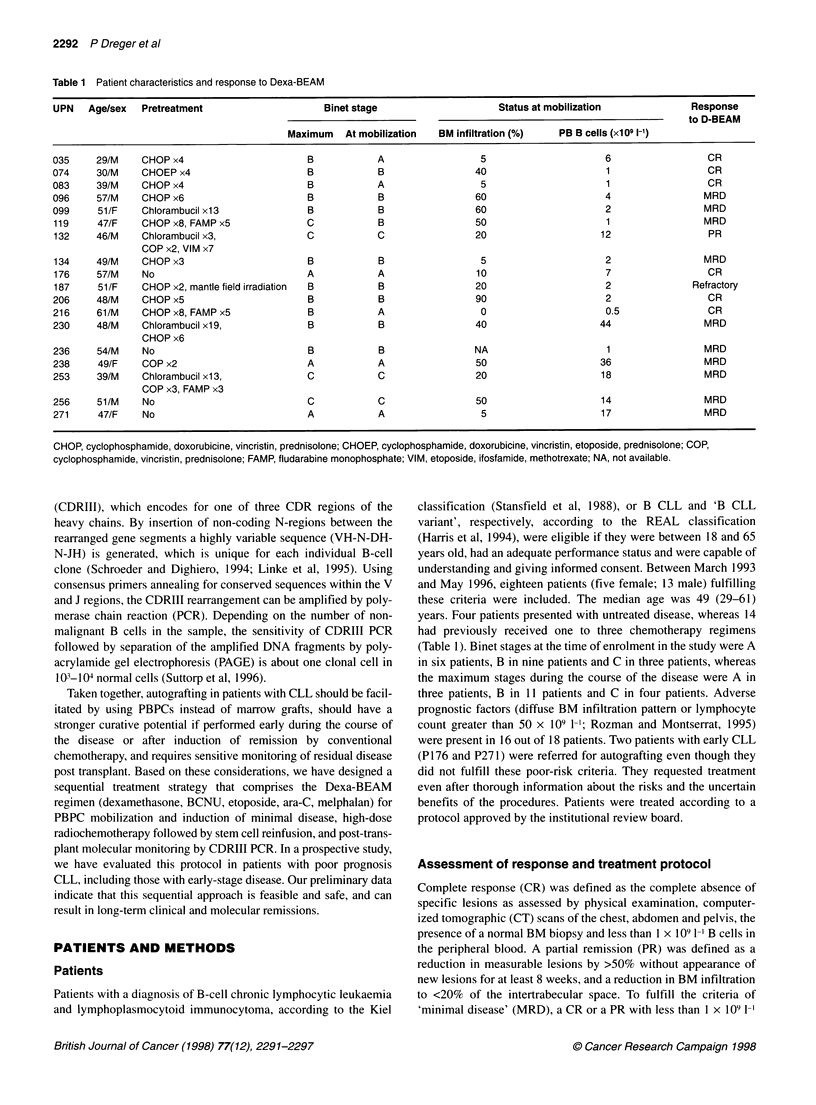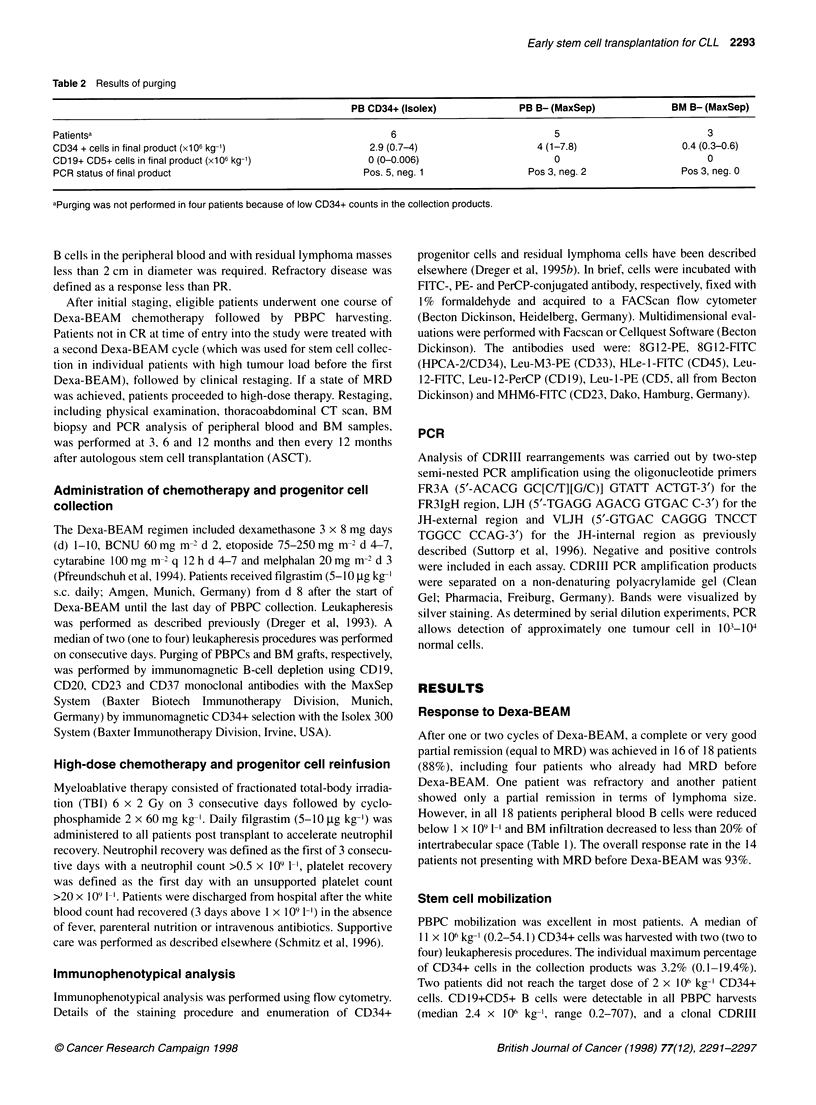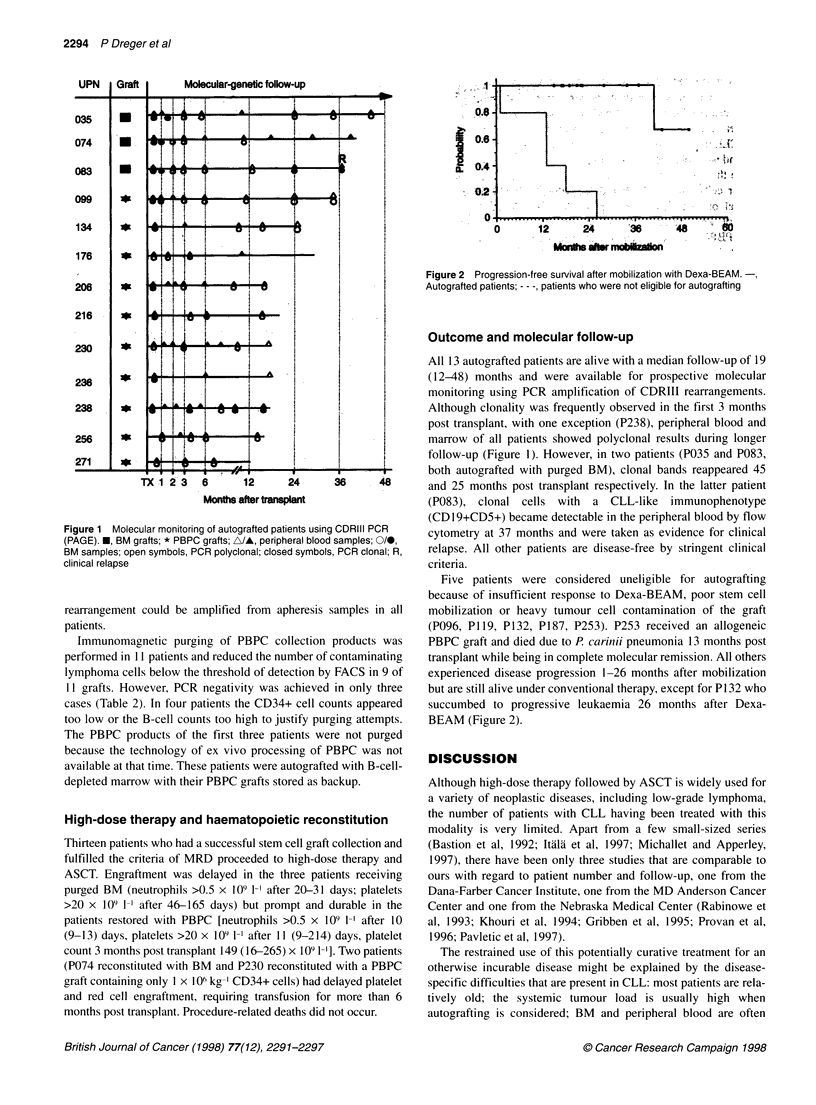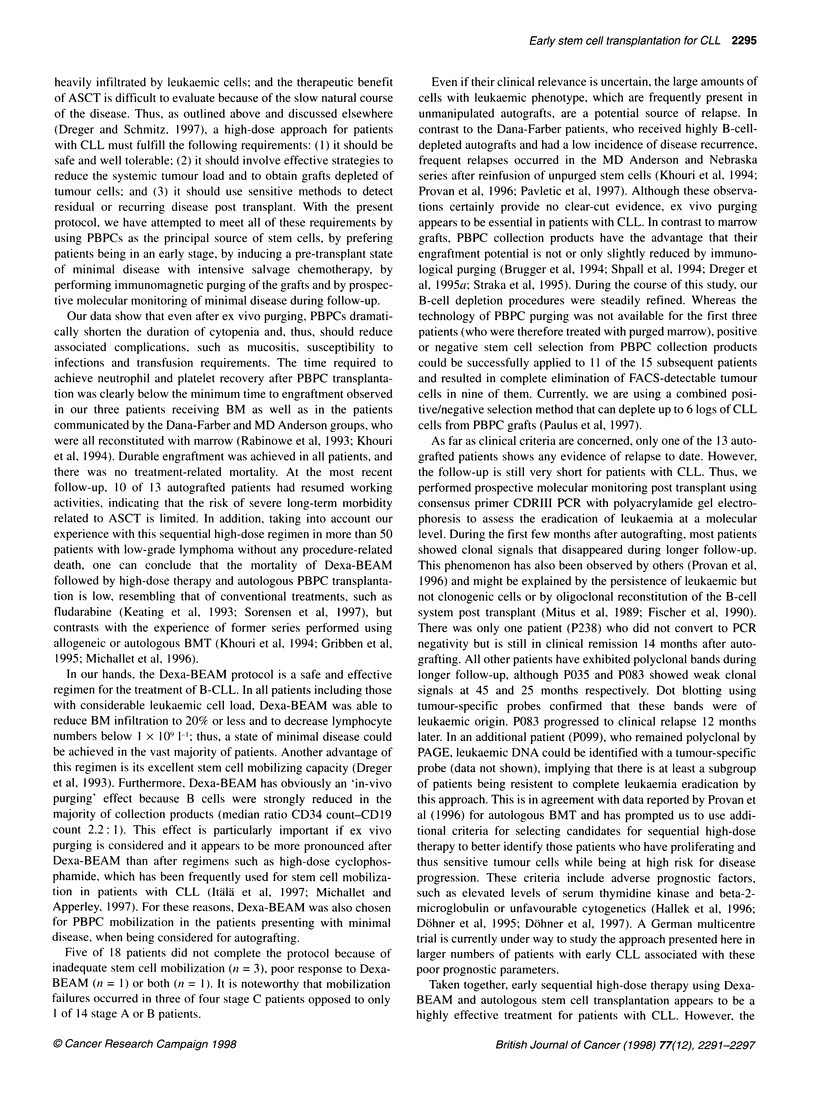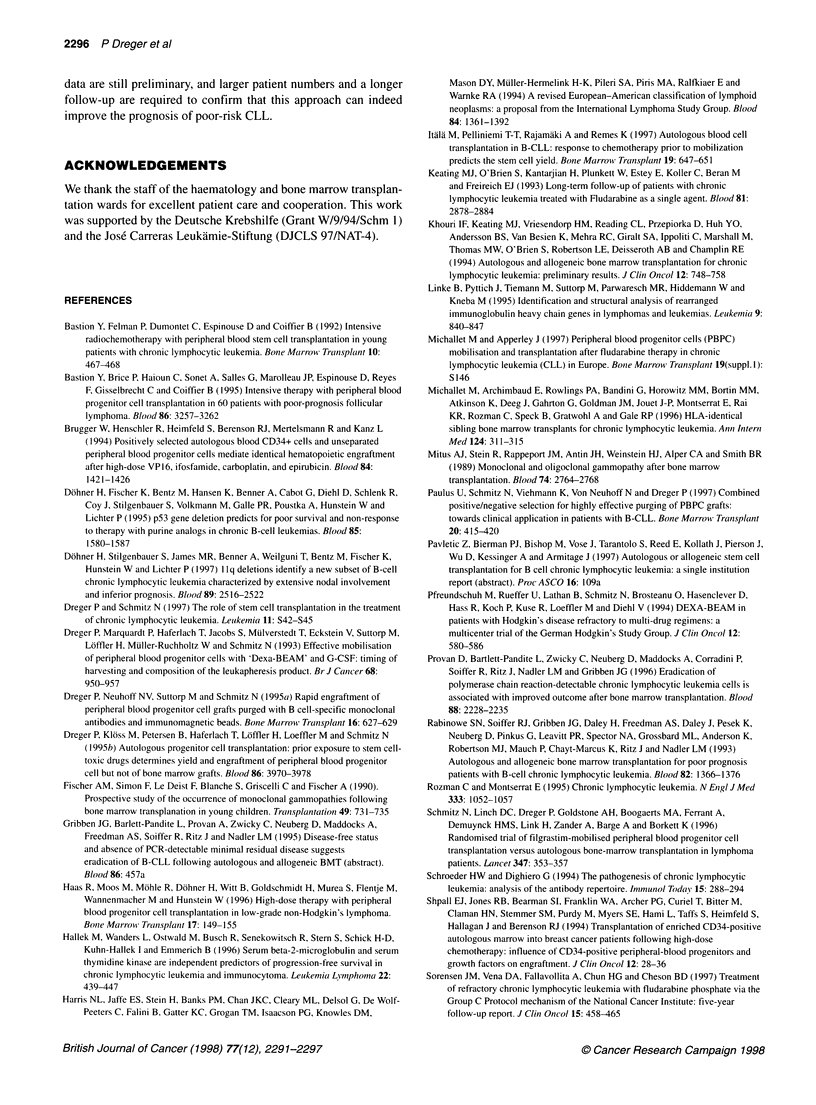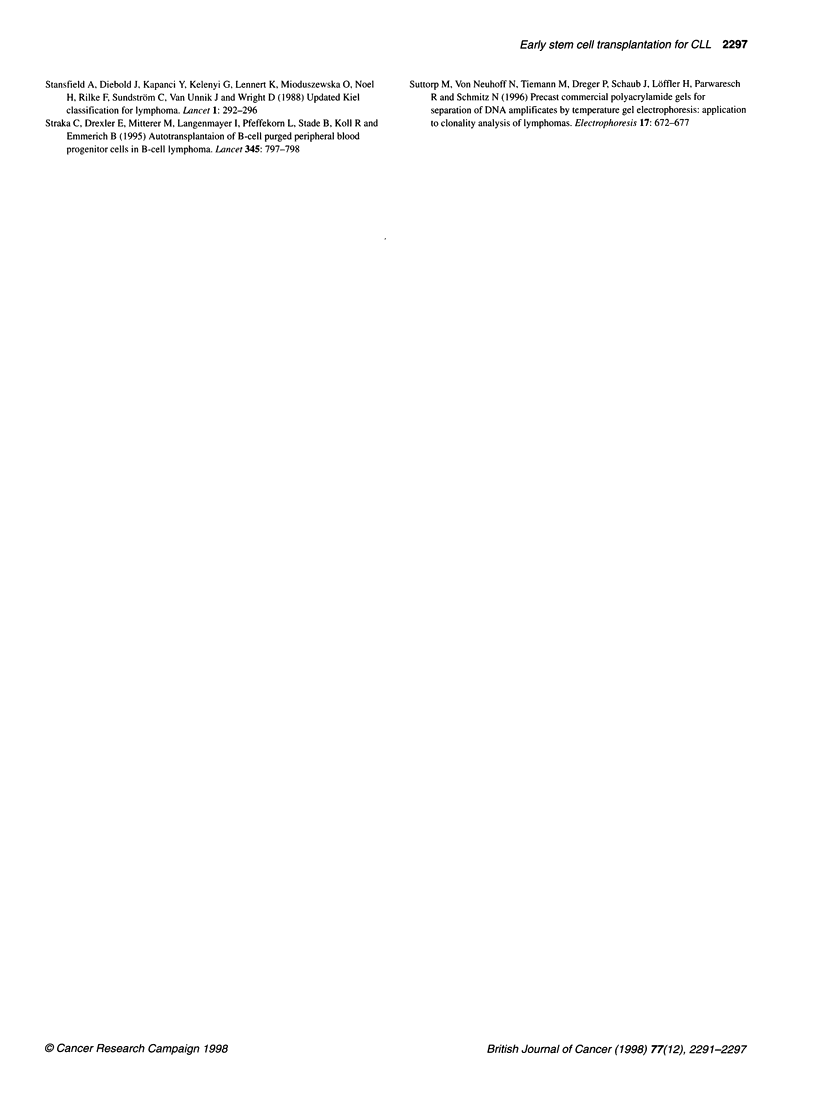# Early stem cell transplantation for chronic lymphocytic leukaemia: a chance for cure?

**DOI:** 10.1038/bjc.1998.381

**Published:** 1998-06

**Authors:** P. Dreger, N. von Neuhoff, R. Kuse, R. Sonnen, B. Glass, L. Uharek, R. Schoch, H. LÃ¶ffler, N. Schmitz

**Affiliations:** Second Department of Medicine, University of Kiel, Germany.

## Abstract

B-cell chronic lymphocytic leukaemia (CLL) cannot be cured by conventional therapy. To improve the prognosis of patients with CLL, we have designed a sequential treatment strategy that comprises intensive chemotherapy for mobilization of peripheral blood progenitor cells (PBPCs) and induction of minimal disease, followed by high-dose radiochemotherapy with stem cell reinfusion and post-transplant molecular monitoring by polymerase chain reaction (PCR) amplification of the complementary determining region III (CDRIII) gene. In a prospective study, we have evaluated this protocol in 18 patients with CLL, also including early stages of the disease. The median age was 49 (29-61) years; Binet stages were A, six; B, nine; and C, three. Adverse prognostic factors [high lymphocyte count and/or diffuse bone marrow (BM) infiltration] were present in 16 out of 18 patients. All patients showed a clone-specific molecular marker as demonstrated by PCR amplification of CDRIII rearrangements. For stem cell mobilization and reduction of tumour load, one to two cycles of Dexa-BEAM chemotherapy were administered, resulting in minimal disease (circulating lymphoma cells <1 x 10(9) l(-1); BM infiltration <20%; lymphomas <2 cm) in 16 out of 18 patients, including four patients who already had minimal disease before Dexa-BEAM. Stem cell harvesting was successful in 14 patients. All grafts [three BM, 11 peripheral blood (PB)] were purged from leukaemic cells using immunomagnetic methods. Thirteen patients having achieved minimal disease were reinfused with purged autologous stem cells (ASC) after preparation with total body irradiation and cyclophosphamide. Engraftment was delayed in patients receiving BM (n = 3) but prompt [neutrophils >0.5 x 10(9) l(-1) after 10 (9-13) days, platelets >20 x 10(9) l(-1) after 11 (9-214) days] in patients restored with PBPCs (n = 10). Procedure-related deaths did not occur. Although the results of CDRIII PCR suggest persistence or recurrence of the leukaemic clone in at least three cases, to date only one patient has relapsed, whereas all others survive without clinical evidence of disease with a maximum follow-up of 48 months. We conclude that sequential high-dose therapy using Dexa-BEAM and autologous stem cell transplantation is a safe and highly effective treatment for patients with CLL. However, a longer follow-up is needed to assess whether definite cures can be achieved using this strategy.


British Journal of Cancer (1998) 77(12), 2291–2297
© 1998 Cancer Research Campaign

### Early stem cell transplantation for chronic lymphocytic leukaemia: a chance for cure?

**P Dreger[1], N von Neuhoff[1], R Kuse[2], R Sonnen[2], B Glass[1], L Uharek[1], R Schoch[1], H Löffler[1] and N Schmitz[1]**

[1]Second Department of Medicine, University of Kiel, Kiel; [2]Department of Hematology, Allgemeines Krankenhaus St Georg, Hamburg, Germany

**Summary** B-cell chronic lymphocytic leukaemia (CLL) cannot be cured by conventional therapy. To improve the prognosis of patients with CLL, we have designed a sequential treatment strategy that comprises intensive chemotherapy for mobilization of peripheral blood progenitor cells (PBPCs) and induction of minimal disease, followed by high-dose radiochemotherapy with stem cell reinfusion and post-transplant molecular monitoring by polymerase chain reaction (PCR) amplification of the complementary determining region III (CDRIII) gene. In a prospective study, we have evaluated this protocol in 18 patients with CLL, also including early stages of the disease. The median age was 49 (29–61) years; Binet stages were A, six; B, nine; and C, three. Adverse prognostic factors [high lymphocyte count and/or diffuse bone marrow (BM) infiltration] were present in 16 out of 18 patients. All patients showed a clone-specific molecular marker as demonstrated by PCR amplification of CDRIII rearrangements. For stem cell mobilization and reduction of tumour load, one to two cycles of Dexa-BEAM chemotherapy were administered, resulting in minimal disease (circulating lymphoma cells $<1 \times 10^9$ $l^{-1}$; BM infiltration <20%; lymphomas <2 cm) in 16 out of 18 patients, including four patients who already had minimal disease before Dexa-BEAM. Stem cell harvesting was successful in 14 patients. All grafts [three BM, 11 peripheral blood (PB)] were purged from leukaemic cells using immunomagnetic methods. Thirteen patients having achieved minimal disease were reinfused with purged autologous stem cells (ASC) after preparation with total body irradiation and cyclophosphamide. Engraftment was delayed in patients receiving BM ($n$ = 3) but prompt [neutrophils $>0.5 \times 10^9$ $l^{-1}$ after 10 (9–13) days, platelets $>20 \times 10^9$ $l^{-1}$ after 11 (9–214) days] in patients restored with PBPCs ($n$ = 10). Procedure-related deaths did not occur. Although the results of CDRIII PCR suggest persistence or recurrence of the leukaemic clone in at least three cases, to date only one patient has relapsed, whereas all others survive without clinical evidence of disease with a maximum follow-up of 48 months. We conclude that sequential high-dose therapy using Dexa-BEAM and autologous stem cell transplantation is a safe and highly effective treatment for patients with CLL. However, a longer follow-up is needed to assess whether definite cures can be achieved using this strategy.

**Keywords:** chronic lymphocytic leukaemia; therapy; stem cell transplantation; polymerase chain reaction

B-cell chronic lymphocytic leukaemia (CLL) is not curable with conventional chemotherapy. Although the natural course of the disease is often indolent, a considerable reduction in survival time must be expected if adverse prognostic factors, such as high leucocyte count, diffuse bone marrow infiltration, short lymphocyte doubling time, unfavourable cytogenetics or advanced stage, are present (Rozman and Montserrat, 1995). Thus, in particular for younger patients, new treatment modalities are required that have the potential to provide long-term remissions or to completely eradicate the disease. Preliminary reports suggest that myeloablative radiochemotherapy with subsequent autologous bone marrow transplantation (BMT) may have curative potential in CLL (Rabinowe et al, 1993; Khouri et al, 1994; Provan et al, 1996).

Wider use of autologous BMT for treatment of CLL has been hindered by a number of concerns. First, autografts from patients with CLL are frequently contaminated with significant amounts of leukaemic cells, necessitating ex vivo tumour cell depletion ('purging') of the graft to reduce potential sources of relapse. In addition, CLL is a disease of the elderly, rendering myeloablative radiochemotherapy with prolonged cytopenias, as usually seen after transplantation of purged marrow, particularly difficult. The use of peripheral blood progenitor cells (PBPCs) instead of BM can shorten the duration of post-transplant aplasia dramatically, especially if ex vivo purged autografts are compared (Schmitz et al, 1996; Dreger et al, 1995*a*) and should thus be particularly beneficial in patients with CLL.

Second, relapses have been observed after BMT, especially in those patients who had a large tumour load before high-dose therapy (Khouri et al, 1994), suggesting that – similar to other haematological neoplasias – achievement of a state of minimal disease before autografting is an important prerequisite for a favourable outcome (Bastion et al, 1995; Haas et al, 1996). Therefore it might be advisable (1) to consider high-dose therapy early during the course of the disease and (2) to reduce the leukaemic burden by cytoreductive chemotherapy before transplant.

Finally, in the patients remaining in clinical remission during the early post-transplant phase, complete eradication of CLL is impossible to assess using clinical criteria or conventional haematological methods, implying that sensitive immunological or molecular markers are needed for adequate monitoring of minimal residual disease. In patients with CLL, molecular-genetic monitoring can be performed using the immunoglobulin heavy-chain (IgH) gene as a disease-specific marker. During B-cell maturation, three different germ-line gene segment families (variable, V; diversity, D; joining, J) of the IgH gene undergo specific rearrangements. One result of this process is the third complementary determining region

*Received 1 September 1997*
*Revised 8 December 1997*
*Accepted 9 December 1997*

*Correspondence to:* P Dreger, Second Department of Medicine, Chemnitzstr. 33, 24116 Kiel, Germany

**Table 1** Patient characteristics and response to Dexa-BEAM

| UPN | Age/sex | Pretreatment | Binet stage | | Status at mobilization | | Response to D-BEAM |
|---|---|---|---|---|---|---|---|
| | | | Maximum | At mobilization | BM infiltration (%) | PB B cells ($\times 10^9$ $l^{-1}$) | |
| 035 | 29/M | CHOP ×4 | B | A | 5 | 6 | CR |
| 074 | 30/M | CHOEP ×4 | B | B | 40 | 1 | CR |
| 083 | 39/M | CHOP ×4 | B | A | 5 | 1 | CR |
| 096 | 57/M | CHOP ×6 | B | B | 60 | 4 | MRD |
| 099 | 51/F | Chlorambucil ×13 | B | B | 60 | 2 | MRD |
| 119 | 47/F | CHOP ×8, FAMP ×5 | C | B | 50 | 1 | MRD |
| 132 | 46/M | Chlorambucil ×3, COP ×2, VIM ×7 | C | C | 20 | 12 | PR |
| 134 | 49/M | CHOP ×3 | B | B | 5 | 2 | MRD |
| 176 | 57/M | No | A | A | 10 | 7 | CR |
| 187 | 51/F | CHOP ×2, mantle field irradiation | B | B | 20 | 2 | Refractory |
| 206 | 48/M | CHOP ×5 | B | B | 90 | 2 | CR |
| 216 | 61/M | CHOP ×8, FAMP ×5 | B | A | 0 | 0.5 | CR |
| 230 | 48/M | Chlorambucil ×19, CHOP ×6 | B | B | 40 | 44 | MRD |
| 236 | 54/M | No | B | B | NA | 1 | MRD |
| 238 | 49/F | COP ×2 | A | A | 50 | 36 | MRD |
| 253 | 39/M | Chlorambucil ×13, COP ×3, FAMP ×3 | C | C | 20 | 18 | MRD |
| 256 | 51/M | No | C | C | 50 | 14 | MRD |
| 271 | 47/F | No | A | A | 5 | 17 | MRD |

CHOP, cyclophosphamide, doxorubicine, vincristin, prednisolone; CHOEP, cyclophosphamide, doxorubicine, vincristin, etoposide, prednisolone; COP, cyclophosphamide, vincristin, prednisolone; FAMP, fludarabine monophosphate; VIM, etoposide, ifosfamide, methotrexate; NA, not available.

(CDRIII), which encodes for one of three CDR regions of the heavy chains. By insertion of non-coding N-regions between the rearranged gene segments a highly variable sequence (VH-N-DH-N-JH) is generated, which is unique for each individual B-cell clone (Schroeder and Dighiero, 1994; Linke et al, 1995). Using consensus primers annealing for conserved sequences within the V and J regions, the CDRIII rearrangement can be amplified by polymerase chain reaction (PCR). Depending on the number of non-malignant B cells in the sample, the sensitivity of CDRIII PCR followed by separation of the amplified DNA fragments by polyacrylamide gel electrophoresis (PAGE) is about one clonal cell in $10^3$–$10^4$ normal cells (Suttorp et al, 1996).

Taken together, autografting in patients with CLL should be facilitated by using PBPCs instead of marrow grafts, should have a stronger curative potential if performed early during the course of the disease or after induction of remission by conventional chemotherapy, and requires sensitive monitoring of residual disease post transplant. Based on these considerations, we have designed a sequential treatment strategy that comprises the Dexa-BEAM regimen (dexamethasone, BCNU, etoposide, ara-C, melphalan) for PBPC mobilization and induction of minimal disease, high-dose radiochemotherapy followed by stem cell reinfusion, and post-transplant molecular monitoring by CDRIII PCR. In a prospective study, we have evaluated this protocol in patients with poor prognosis CLL, including those with early-stage disease. Our preliminary data indicate that this sequential approach is feasible and safe, and can result in long-term clinical and molecular remissions.

### PATIENTS AND METHODS

### Patients

Patients with a diagnosis of B-cell chronic lymphocytic leukaemia and lymphoplasmocytoid immunocytoma, according to the Kiel classification (Stansfield et al, 1988), or B CLL and 'B CLL variant', respectively, according to the REAL classification (Harris et al, 1994), were eligible if they were between 18 and 65 years old, had an adequate performance status and were capable of understanding and giving informed consent. Between March 1993 and May 1996, eighteen patients (five female; 13 male) fulfilling these criteria were included. The median age was 49 (29–61) years. Four patients presented with untreated disease, whereas 14 had previously received one to three chemotherapy regimens (Table 1). Binet stages at the time of enrolment in the study were A in six patients, B in nine patients and C in three patients, whereas the maximum stages during the course of the disease were A in three patients, B in 11 patients and C in four patients. Adverse prognostic factors (diffuse BM infiltration pattern or lymphocyte count greater than $50 \times 10^9$ $l^{-1}$; Rozman and Montserrat, 1995) were present in 16 out of 18 patients. Two patients with early CLL (P176 and P271) were referred for autografting even though they did not fulfill these poor-risk criteria. They requested treatment even after thorough information about the risks and the uncertain benefits of the procedures. Patients were treated according to a protocol approved by the institutional review board.

### Assessment of response and treatment protocol

Complete response (CR) was defined as the complete absence of specific lesions as assessed by physical examination, computerized tomographic (CT) scans of the chest, abdomen and pelvis, the presence of a normal BM biopsy and less than $1 \times 10^9$ $l^{-1}$ B cells in the peripheral blood. A partial remission (PR) was defined as a reduction in measurable lesions by >50% without appearance of new lesions for at least 8 weeks, and a reduction in BM infiltration to <20% of the intertrabecular space. To fulfill the criteria of 'minimal disease' (MRD), a CR or a PR with less than $1 \times 10^9$ $l^{-1}$

 © Cancer Research Campaign 1998

**Table 2** Results of purging

| | PB CD34+ (Isolex) | PB B– (MaxSep) | BM B– (MaxSep) |
|---|---|---|---|
| Patients[a] | 6 | 5 | 3 |
| CD34 + cells in final product ($\times 10^6$ kg$^{-1}$) | 2.9 (0.7–4) | 4 (1–7.8) | 0.4 (0.3–0.6) |
| CD19+ CD5+ cells in final product ($\times 10^6$ kg$^{-1}$) | 0 (0–0.006) | 0 | 0 |
| PCR status of final product | Pos. 5, neg. 1 | Pos 3, neg. 2 | Pos 3, neg. 0 |

[a]Purging was not performed in four patients because of low CD34+ counts in the collection products.

B cells in the peripheral blood and with residual lymphoma masses less than 2 cm in diameter was required. Refractory disease was defined as a response less than PR.

After initial staging, eligible patients underwent one course of Dexa-BEAM chemotherapy followed by PBPC harvesting. Patients not in CR at time of entry into the study were treated with a second Dexa-BEAM cycle (which was used for stem cell collection in individual patients with high tumour load before the first Dexa-BEAM), followed by clinical restaging. If a state of MRD was achieved, patients proceeded to high-dose therapy. Restaging, including physical examination, thoracoabdominal CT scan, BM biopsy and PCR analysis of peripheral blood and BM samples, was performed at 3, 6 and 12 months and then every 12 months after autologous stem cell transplantation (ASCT).

### Administration of chemotherapy and progenitor cell collection

The Dexa-BEAM regimen included dexamethasone 3 × 8 mg days (d) 1–10, BCNU 60 mg m$^{-2}$ d 2, etoposide 75–250 mg m$^{-2}$ d 4–7, cytarabine 100 mg m$^{-2}$ q 12 h d 4–7 and melphalan 20 mg m$^{-2}$ d 3 (Pfreundschuh et al, 1994). Patients received filgrastim (5–10 μg kg$^{-1}$ s.c. daily; Amgen, Munich, Germany) from d 8 after the start of Dexa-BEAM until the last day of PBPC collection. Leukapheresis was performed as described previously (Dreger et al, 1993). A median of two (one to four) leukapheresis procedures was performed on consecutive days. Purging of PBPCs and BM grafts, respectively, was performed by immunomagnetic B-cell depletion using CD19, CD20, CD23 and CD37 monoclonal antibodies with the MaxSep System (Baxter Biotech Immunotherapy Division, Munich, Germany) by immunomagnetic CD34+ selection with the Isolex 300 System (Baxter Immunotherapy Division, Irvine, USA).

### High-dose chemotherapy and progenitor cell reinfusion

Myeloablative therapy consisted of fractionated total-body irradiation (TBI) 6 × 2 Gy on 3 consecutive days followed by cyclophosphamide 2 × 60 mg kg$^{-1}$. Daily filgrastim (5–10 μg kg$^{-1}$) was administered to all patients post transplant to accelerate neutrophil recovery. Neutrophil recovery was defined as the first of 3 consecutive days with a neutrophil count >0.5 × $10^9$ l$^{-1}$, platelet recovery was defined as the first day with an unsupported platelet count >20 × $10^9$ l$^{-1}$. Patients were discharged from hospital after the white blood count had recovered (3 days above 1 × $10^9$ l$^{-1}$) in the absence of fever, parenteral nutrition or intravenous antibiotics. Supportive care was performed as described elsewhere (Schmitz et al, 1996).

### Immunophenotypical analysis

Immunophenotypical analysis was performed using flow cytometry. Details of the staining procedure and enumeration of CD34+ progenitor cells and residual lymphoma cells have been described elsewhere (Dreger et al, 1995*b*). In brief, cells were incubated with FITC-, PE- and PerCP-conjugated antibody, respectively, fixed with 1% formaldehyde and acquired to a FACScan flow cytometer (Becton Dickinson, Heidelberg, Germany). Multidimensional evaluations were performed with Facscan or Cellquest Software (Becton Dickinson). The antibodies used were: 8G12-PE, 8G12-FITC (HPCA-2/CD34), Leu-M3-PE (CD33), HLe-1-FITC (CD45), Leu-12-FITC, Leu-12-PerCP (CD19), Leu-1-PE (CD5, all from Becton Dickinson) and MHM6-FITC (CD23, Dako, Hamburg, Germany).

### PCR

Analysis of CDRIII rearrangements was carried out by two-step semi-nested PCR amplification using the oligonucleotide primers FR3A (5′-ACACG GC[C/T][G/C)] GTATT ACTGT-3′) for the FR3IgH region, LJH (5′-TGAGG AGACG GTGAC C-3′) for the JH-external region and VLJH (5′-GTGAC CAGGG TNCCT TGGCC CCAG-3′) for the JH-internal region as previously described (Suttorp et al, 1996). Negative and positive controls were included in each assay. CDRIII PCR amplification products were separated on a non-denaturing polyacrylamide gel (Clean Gel; Pharmacia, Freiburg, Germany). Bands were visualized by silver staining. As determined by serial dilution experiments, PCR allows detection of approximately one tumour cell in $10^3$–$10^4$ normal cells.

### RESULTS

### Response to Dexa-BEAM

After one or two cycles of Dexa-BEAM, a complete or very good partial remission (equal to MRD) was achieved in 16 of 18 patients (88%), including four patients who already had MRD before Dexa-BEAM. One patient was refractory and another patient showed only a partial remission in terms of lymphoma size. However, in all 18 patients peripheral blood B cells were reduced below 1 × $10^9$ l$^{-1}$ and BM infiltration decreased to less than 20% of intertrabecular space (Table 1). The overall response rate in the 14 patients not presenting with MRD before Dexa-BEAM was 93%.

### Stem cell mobilization

PBPC mobilization was excellent in most patients. A median of 11 × $10^6$ kg$^{-1}$ (0.2–54.1) CD34+ cells was harvested with two (two to four) leukapheresis procedures. The individual maximum percentage of CD34+ cells in the collection products was 3.2% (0.1–19.4%). Two patients did not reach the target dose of 2 × $10^6$ kg$^{-1}$ CD34+ cells. CD19+CD5+ B cells were detectable in all PBPC harvests (median 2.4 × $10^6$ kg$^{-1}$, range 0.2–707), and a clonal CDRIII

© Cancer Research Campaign 1998

**Figure 1** Molecular monitoring of autografted patients using CDRIII PCR (PAGE). ■, BM grafts; * PBPC grafts; △/▲, peripheral blood samples; ○/●, BM samples; open symbols, PCR polyclonal; closed symbols, PCR clonal; R, clinical relapse rearrangement could be amplified from apheresis samples in all patients.

Immunomagnetic purging of PBPC collection products was performed in 11 patients and reduced the number of contaminating lymphoma cells below the threshold of detection by FACS in 9 of 11 grafts. However, PCR negativity was achieved in only three cases (Table 2). In four patients the CD34+ cell counts appeared too low or the B-cell counts too high to justify purging attempts. The PBPC products of the first three patients were not purged because the technology of ex vivo processing of PBPC was not available at that time. These patients were autografted with B-cell-depleted marrow with their PBPC grafts stored as backup.

### High-dose therapy and haematopoietic reconstitution

Thirteen patients who had a successful stem cell graft collection and fulfilled the criteria of MRD proceeded to high-dose therapy and ASCT. Engraftment was delayed in the three patients receiving purged BM (neutrophils $>0.5 \times 10^9$ $l^{-1}$ after 20–31 days; platelets $>20 \times 10^9$ $l^{-1}$ after 46–165 days) but prompt and durable in the patients restored with PBPC [neutrophils $>0.5 \times 10^9$ $l^{-1}$ after 10 (9–13) days, platelets $>20 \times 10^9$ $l^{-1}$ after 11 (9–214) days, platelet count 3 months post transplant 149 (16–265) $\times 10^9$ $l^{-1}$]. Two patients (P074 reconstituted with BM and P230 reconstituted with a PBPC graft containing only $1 \times 10^6$ $kg^{-1}$ CD34+ cells) had delayed platelet and red cell engraftment, requiring transfusion for more than 6 months post transplant. Procedure-related deaths did not occur.

**Figure 2** Progression-free survival after mobilization with Dexa-BEAM. —, Autografted patients; - - -, patients who were not eligible for autografting

### Outcome and molecular follow-up

All 13 autografted patients are alive with a median follow-up of 19 (12–48) months and were available for prospective molecular monitoring using PCR amplification of CDRIII rearrangements. Although clonality was frequently observed in the first 3 months post transplant, with one exception (P238), peripheral blood and marrow of all patients showed polyclonal results during longer follow-up (Figure 1). However, in two patients (P035 and P083, both autografted with purged BM), clonal bands reappeared 45 and 25 months post transplant respectively. In the latter patient (P083), clonal cells with a CLL-like immunophenotype (CD19+CD5+) became detectable in the peripheral blood by flow cytometry at 37 months and were taken as evidence for clinical relapse. All other patients are disease-free by stringent clinical criteria.

Five patients were considered uneligible for autografting because of insufficient response to Dexa-BEAM, poor stem cell mobilization or heavy tumour cell contamination of the graft (P096, P119, P132, P187, P253). P253 received an allogeneic PBPC graft and died due to *P. carinii* pneumonia 13 months post transplant while being in complete molecular remission. All others experienced disease progression 1–26 months after mobilization but are still alive under conventional therapy, except for P132 who succumbed to progressive leukaemia 26 months after Dexa-BEAM (Figure 2).

### DISCUSSION

Although high-dose therapy followed by ASCT is widely used for a variety of neoplastic diseases, including low-grade lymphoma, the number of patients with CLL having been treated with this modality is very limited. Apart from a few small-sized series (Bastion et al, 1992; Itälä et al, 1997; Michallet and Apperley, 1997), there have been only three studies that are comparable to ours with regard to patient number and follow-up, one from the Dana-Farber Cancer Institute, one from the MD Anderson Cancer Center and one from the Nebraska Medical Center (Rabinowe et al, 1993; Khouri et al, 1994; Gribben et al, 1995; Provan et al, 1996; Pavletic et al, 1997).

The restrained use of this potentially curative treatment for an otherwise incurable disease might be explained by the disease-specific difficulties that are present in CLL: most patients are relatively old; the systemic tumour load is usually high when autografting is considered; BM and peripheral blood are often

*© Cancer Research Campaign 1998*

heavily infiltrated by leukaemic cells; and the therapeutic benefit of ASCT is difficult to evaluate because of the slow natural course of the disease. Thus, as outlined above and discussed elsewhere (Dreger and Schmitz, 1997), a high-dose approach for patients with CLL must fulfill the following requirements: (1) it should be safe and well tolerable; (2) it should involve effective strategies to reduce the systemic tumour load and to obtain grafts depleted of tumour cells; and (3) it should use sensitive methods to detect residual or recurring disease post transplant. With the present protocol, we have attempted to meet all of these requirements by using PBPCs as the principal source of stem cells, by prefering patients being in an early stage, by inducing a pre-transplant state of minimal disease with intensive salvage chemotherapy, by performing immunomagnetic purging of the grafts and by prospective molecular monitoring of minimal disease during follow-up.

Our data show that even after ex vivo purging, PBPCs dramatically shorten the duration of cytopenia and, thus, should reduce associated complications, such as mucositis, susceptibility to infections and transfusion requirements. The time required to achieve neutrophil and platelet recovery after PBPC transplantation was clearly below the minimum time to engraftment observed in our three patients receiving BM as well as in the patients communicated by the Dana-Farber and MD Anderson groups, who were all reconstituted with marrow (Rabinowe et al, 1993; Khouri et al, 1994). Durable engraftment was achieved in all patients, and there was no treatment-related mortality. At the most recent follow-up, 10 of 13 autografted patients had resumed working activities, indicating that the risk of severe long-term morbidity related to ASCT is limited. In addition, taking into account our experience with this sequential high-dose regimen in more than 50 patients with low-grade lymphoma without any procedure-related death, one can conclude that the mortality of Dexa-BEAM followed by high-dose therapy and autologous PBPC transplantation is low, resembling that of conventional treatments, such as fludarabine (Keating et al, 1993; Sorensen et al, 1997), but contrasts with the experience of former series performed using allogeneic or autologous BMT (Khouri et al, 1994; Gribben et al, 1995; Michallet et al, 1996).

In our hands, the Dexa-BEAM protocol is a safe and effective regimen for the treatment of B-CLL. In all patients including those with considerable leukaemic cell load, Dexa-BEAM was able to reduce BM infiltration to 20% or less and to decrease lymphocyte numbers below $1 \times 10^9\ l^{-1}$; thus, a state of minimal disease could be achieved in the vast majority of patients. Another advantage of this regimen is its excellent stem cell mobilizing capacity (Dreger et al, 1993). Furthermore, Dexa-BEAM has obviously an 'in-vivo purging' effect because B cells were strongly reduced in the majority of collection products (median ratio CD34 count–CD19 count 2.2 : 1). This effect is particularly important if ex vivo purging is considered and it appears to be more pronounced after Dexa-BEAM than after regimens such as high-dose cyclophosphamide, which has been frequently used for stem cell mobilization in patients with CLL (Itälä et al, 1997; Michallet and Apperley, 1997). For these reasons, Dexa-BEAM was also chosen for PBPC mobilization in the patients presenting with minimal disease, when being considered for autografting.

Five of 18 patients did not complete the protocol because of inadequate stem cell mobilization ($n$ = 3), poor response to Dexa-BEAM ($n$ = 1) or both ($n$ = 1). It is noteworthy that mobilization failures occurred in three of four stage C patients opposed to only 1 of 14 stage A or B patients.

Even if their clinical relevance is uncertain, the large amounts of cells with leukaemic phenotype, which are frequently present in unmanipulated autografts, are a potential source of relapse. In contrast to the Dana-Farber patients, who received highly B-cell-depleted autografts and had a low incidence of disease recurrence, frequent relapses occurred in the MD Anderson and Nebraska series after reinfusion of unpurged stem cells (Khouri et al, 1994; Provan et al, 1996; Pavletic et al, 1997). Although these observations certainly provide no clear-cut evidence, ex vivo purging appears to be essential in patients with CLL. In contrast to marrow grafts, PBPC collection products have the advantage that their engraftment potential is not or only slightly reduced by immunological purging (Brugger et al, 1994; Shpall et al, 1994; Dreger et al, 1995*a*; Straka et al, 1995). During the course of this study, our B-cell depletion procedures were steadily refined. Whereas the technology of PBPC purging was not available for the first three patients (who were therefore treated with purged marrow), positive or negative stem cell selection from PBPC collection products could be successfully applied to 11 of the 15 subsequent patients and resulted in complete elimination of FACS-detectable tumour cells in nine of them. Currently, we are using a combined positive/negative selection method that can deplete up to 6 logs of CLL cells from PBPC grafts (Paulus et al, 1997).

As far as clinical criteria are concerned, only one of the 13 autografted patients shows any evidence of relapse to date. However, the follow-up is still very short for patients with CLL. Thus, we performed prospective molecular monitoring post transplant using consensus primer CDRIII PCR with polyacrylamide gel electrophoresis to assess the eradication of leukaemia at a molecular level. During the first few months after autografting, most patients showed clonal signals that disappeared during longer follow-up. This phenomenon has also been observed by others (Provan et al, 1996) and might be explained by the persistence of leukaemic but not clonogenic cells or by oligoclonal reconstitution of the B-cell system post transplant (Mitus et al, 1989; Fischer et al, 1990). There was only one patient (P238) who did not convert to PCR negativity but is still in clinical remission 14 months after autografting. All other patients have exhibited polyclonal bands during longer follow-up, although P035 and P083 showed weak clonal signals at 45 and 25 months respectively. Dot blotting using tumour-specific probes confirmed that these bands were of leukaemic origin. P083 progressed to clinical relapse 12 months later. In an additional patient (P099), who remained polyclonal by PAGE, leukaemic DNA could be identified with a tumour-specific probe (data not shown), implying that there is at least a subgroup of patients being resistent to complete leukaemia eradication by this approach. This is in agreement with data reported by Provan et al (1996) for autologous BMT and has prompted us to use additional criteria for selecting candidates for sequential high-dose therapy to better identify those patients who have proliferating and thus sensitive tumour cells while being at high risk for disease progression. These criteria include adverse prognostic factors, such as elevated levels of serum thymidine kinase and beta-2-microglobulin or unfavourable cytogenetics (Hallek et al, 1996; Döhner et al, 1995; Döhner et al, 1997). A German multicentre trial is currently under way to study the approach presented here in larger numbers of patients with early CLL associated with these poor prognostic parameters.

Taken together, early sequential high-dose therapy using Dexa-BEAM and autologous stem cell transplantation appears to be a highly effective treatment for patients with CLL. However, the

© Cancer Research Campaign 1998

data are still preliminary, and larger patient numbers and a longer follow-up are required to confirm that this approach can indeed improve the prognosis of poor-risk CLL.

### ACKNOWLEDGEMENTS

We thank the staff of the haematology and bone marrow transplantation wards for excellent patient care and cooperation. This work was supported by the Deutsche Krebshilfe (Grant W/9/94/Schm 1) and the José Carreras Leukämie-Stiftung (DJCLS 97/NAT-4).

### REFERENCES

Bastion Y, Felman P, Dumontet C, Espinouse D and Coiffier B (1992) Intensive radiochemotherapy with peripheral blood stem cell transplantation in young patients with chronic lymphocytic leukemia. *Bone Marrow Transplant* **10**: 467–468

Bastion Y, Brice P, Haioun C, Sonet A, Salles G, Marolleau JP, Espinouse D, Reyes F, Gisselbrecht C and Coiffier B (1995) Intensive therapy with peripheral blood progenitor cell transplantation in 60 patients with poor-prognosis follicular lymphoma. *Blood* **86**: 3257–3262

Brugger W, Henschler R, Heimfeld S, Berenson RJ, Mertelsmann R and Kanz L (1994) Positively selected autologous blood CD34+ cells and unseparated peripheral blood progenitor cells mediate identical hematopoietic engraftment after high-dose VP16, ifosfamide, carboplatin, and epirubicin. *Blood* **84**: 1421–1426

Döhner H, Fischer K, Bentz M, Hansen K, Benner A, Cabot G, Diehl D, Schlenk R, Coy J, Stilgenbauer S, Volkmann M, Galle PR, Poustka A, Hunstein W and Lichter P (1995) p53 gene deletion predicts for poor survival and non-response to therapy with purine analogs in chronic B-cell leukemias. *Blood* **85**: 1580–1587

Döhner H, Stilgenbauer S, James MR, Benner A, Weilguni T, Bentz M, Fischer K, Hunstein W and Lichter P (1997) 11q deletions identify a new subset of B-cell chronic lymphocytic leukemia characterized by extensive nodal involvement and inferior prognosis. *Blood* **89**: 2516–2522

Dreger P and Schmitz N (1997) The role of stem cell transplantation in the treatment of chronic lymphocytic leukemia. *Leukemia* **11**: S42–S45

Dreger P, Marquardt P, Haferlach T, Jacobs S, Mülverstedt T, Eckstein V, Suttorp M, Löffler H, Müller-Ruchholtz W and Schmitz N (1993) Effective mobilisation of peripheral blood progenitor cells with 'Dexa-BEAM' and G-CSF: timing of harvesting and composition of the leukapheresis product. *Br J Cancer* **68**: 950–957

Dreger P, Neuhoff NV, Suttorp M and Schmitz N (1995*a*) Rapid engraftment of peripheral blood progenitor cell grafts purged with B cell-specific monoclonal antibodies and immunomagnetic beads. *Bone Marrow Transplant* **16**: 627–629

Dreger P, Klöss M, Petersen B, Haferlach T, Löffler H, Loeffler M and Schmitz N (1995*b*) Autologous progenitor cell transplantation: prior exposure to stem cell-toxic drugs determines yield and engraftment of peripheral blood progenitor cell but not of bone marrow grafts. *Blood* **86**: 3970–3978

Fischer AM, Simon F, Le Deist F, Blanche S, Griscelli C and Fischer A (1990). Prospective study of the occurrence of monoclonal gammopathies following bone marrow transplanation in young children. *Transplantation* **49**: 731–735

Gribben JG, Barlett-Pandite L, Provan A, Zwicky C, Neuberg D, Maddocks A, Freedman AS, Soiffer R, Ritz J and Nadler LM (1995) Disease-free status and absence of PCR-detectable minimal residual disease suggests eradication of B-CLL following autologous and allogeneic BMT (abstract). *Blood* **86**: 457a

Haas R, Moos M, Möhle R, Döhner H, Witt B, Goldschmidt H, Murea S, Flentje M, Wannenmacher M and Hunstein W (1996) High-dose therapy with peripheral blood progenitor cell transplantation in low-grade non-Hodgkin's lymphoma. *Bone Marrow Transplant* **17**: 149–155

Hallek M, Wanders L, Ostwald M, Busch R, Senekowitsch R, Stern S, Schick H-D, Kuhn-Hallek I and Emmerich B (1996) Serum beta-2-microglobulin and serum thymidine kinase are independent predictors of progression-free survival in chronic lymphocytic leukemia and immunocytoma. *Leukemia Lymphoma* **22**: 439–447

Harris NL, Jaffe ES, Stein H, Banks PM, Chan JKC, Cleary ML, Delsol G, De Wolf-Peeters C, Falini B, Gatter KC, Grogan TM, Isaacson PG, Knowles DM, Mason DY, Müller-Hermelink H-K, Pileri SA, Piris MA, Ralfkiaer E and Warnke RA (1994) A revised European–American classification of lymphoid neoplasms: a proposal from the International Lymphoma Study Group. *Blood* **84**: 1361–1392

Itälä M, Pelliniemi T-T, Rajamäki A and Remes K (1997) Autologous blood cell transplantation in B-CLL: response to chemotherapy prior to mobilization predicts the stem cell yield. *Bone Marrow Transplant* **19**: 647–651

Keating MJ, O'Brien S, Kantarjian H, Plunkett W, Estey E, Koller C, Beran M and Freireich EJ (1993) Long-term follow-up of patients with chronic lymphocytic leukemia treated with Fludarabine as a single agent. *Blood* **81**: 2878–2884

Khouri IF, Keating MJ, Vriesendorp HM, Reading CL, Przepiorka D, Huh YO, Andersson BS, Van Besien K, Mehra RC, Giralt SA, Ippoliti C, Marshall M, Thomas MW, O'Brien S, Robertson LE, Deisseroth AB and Champlin RE (1994) Autologous and allogeneic bone marrow transplantation for chronic lymphocytic leukemia: preliminary results. *J Clin Oncol* **12**: 748–758

Linke B, Pyttich J, Tiemann M, Suttorp M, Parwaresch MR, Hiddemann W and Kneba M (1995) Identification and structural analysis of rearranged immunoglobulin heavy chain genes in lymphomas and leukemias. *Leukemia* **9**: 840–847

Michallet M and Apperley J (1997) Peripheral blood progenitor cells (PBPC) mobilisation and transplantation after fludarabine therapy in chronic lymphocytic leukemia (CLL) in Europe. *Bone Marrow Transplant* **19**(suppl.1): S146

Michallet M, Archimbaud E, Rowlings PA, Bandini G, Horowitz MM, Bortin MM, Atkinson K, Deeg J, Gahrton G, Goldman JM, Jouet J-P, Montserrat E, Rai KR, Rozman C, Speck B, Gratwohl A and Gale RP (1996) HLA-identical sibling bone marrow transplants for chronic lymphocytic leukemia. *Ann Intern Med* **124**: 311–315

Mitus AJ, Stein R, Rappeport JM, Antin JH, Weinstein HJ, Alper CA and Smith BR (1989) Monoclonal and oligoclonal gammopathy after bone marrow transplantation. *Blood* **74**: 2764–2768

Paulus U, Schmitz N, Viehmann K, Von Neuhoff N and Dreger P (1997) Combined positive/negative selection for highly effective purging of PBPC grafts: towards clinical application in patients with B-CLL. *Bone Marrow Transplant* **20**: 415–420

Pavletic Z, Bierman PJ, Bishop M, Vose J, Tarantolo S, Reed E, Kollath J, Pierson J, Wu D, Kessinger A and Armitage J (1997) Autologous or allogeneic stem cell transplantation for B cell chronic lymphocytic leukemia: a single institution report (abstract). *Proc ASCO* **16**: 109a

Pfreundschuh M, Rueffer U, Lathan B, Schmitz N, Brosteanu O, Hasenclever D, Hass R, Koch P, Kuse R, Loeffler M and Diehl V (1994) DEXA-BEAM in patients with Hodgkin's disease refractory to multi-drug regimens: a multicenter trial of the German Hodgkin's Study Group. *J Clin Oncol* **12**: 580–586

Provan D, Bartlett-Pandite L, Zwicky C, Neuberg D, Maddocks A, Corradini P, Soiffer R, Ritz J, Nadler LM and Gribben JG (1996) Eradication of polymerase chain reaction-detectable chronic lymphocytic leukemia cells is associated with improved outcome after bone marrow transplantation. *Blood* **88**: 2228–2235

Rabinowe SN, Soiffer RJ, Gribben JG, Daley H, Freedman AS, Daley J, Pesek K, Neuberg D, Pinkus G, Leavitt PR, Spector NA, Grossbard ML, Anderson K, Robertson MJ, Mauch P, Chayt-Marcus K, Ritz J and Nadler LM (1993) Autologous and allogeneic bone marrow transplantation for poor prognosis patients with B-cell chronic lymphocytic leukemia. *Blood* **82**: 1366–1376

Rozman C and Montserrat E (1995) Chronic lymphocytic leukemia. *N Engl J Med* **333**: 1052–1057

Schmitz N, Linch DC, Dreger P, Goldstone AH, Boogaerts MA, Ferrant A, Demuynck HMS, Link H, Zander A, Barge A and Borkett K (1996) Randomised trial of filgrastim-mobilised peripheral blood progenitor cell transplantation versus autologous bone-marrow transplantation in lymphoma patients. *Lancet* **347**: 353–357

Schroeder HW and Dighiero G (1994) The pathogenesis of chronic lymphocytic leukemia: analysis of the antibody repertoire. *Immunol Today* **15**: 288–294

Shpall EJ, Jones RB, Bearman SI, Franklin WA, Archer PG, Curiel T, Bitter M, Claman HN, Stemmer SM, Purdy M, Myers SE, Hami L, Taffs S, Heimfeld S, Hallagan J and Berenson RJ (1994) Transplantation of enriched CD34-positive autologous marrow into breast cancer patients following high-dose chemotherapy: influence of CD34-positive peripheral-blood progenitors and growth factors on engraftment. *J Clin Oncol* **12**: 28–36

Sorensen JM, Vena DA, Fallavollita A, Chun HG and Cheson BD (1997) Treatment of refractory chronic lymphocytic leukemia with fludarabine phosphate via the Group C Protocol mechanism of the National Cancer Institute: five-year follow-up report. *J Clin Oncol* **15**: 458–465

© Cancer Research Campaign 1998

Stansfield A, Diebold J, Kapanci Y, Kelenyi G, Lennert K, Mioduszewska O, Noel H, Rilke F, Sundström C, Van Unnik J and Wright D (1988) Updated Kiel classification for lymphoma. *Lancet* **1**: 292–296

Straka C, Drexler E, Mitterer M, Langenmayer I, Pfeffekorn L, Stade B, Koll R and Emmerich B (1995) Autotransplantaion of B-cell purged peripheral blood progenitor cells in B-cell lymphoma. *Lancet* **345**: 797–798

Suttorp M, Von Neuhoff N, Tiemann M, Dreger P, Schaub J, Löffler H, Parwaresch R and Schmitz N (1996) Precast commercial polyacrylamide gels for separation of DNA amplificates by temperature gel electrophoresis: application to clonality analysis of lymphomas. *Electrophoresis* **17**: 672–677

© Cancer Research Campaign 1998